# Influence of particle size and extraction solvent on antioxidant properties of extracts of tea, ginger, and tea–ginger blend

**DOI:** 10.1002/fsn3.509

**Published:** 2017-08-29

**Authors:** Solomon A. Makanjuola

**Affiliations:** ^1^ Department of Food Science and Technology Federal University of Technology Akure Nigeria; ^2^ BloomMak Scientific Services Gbagada Lagos Nigeria

**Keywords:** antioxidants, ginger, particle size, solvent extraction, tea

## Abstract

The influence of particle size and extraction solvent on the antioxidant properties of aqueous and ethanolic extracts of tea (*Camellia sinensis*), ginger (*Zingiber officinale*), and tea–ginger (2:1) blend was investigated. The powder sizes studied were 0.425, 0.710, and 1.180 mm. Extracts were analyzed for DPPH radical scavenging activity, ABTS radical scavenging activity, total phenol content (TPC), iron chelating activity, total flavonoid content, and peroxide scavenging activity. The powder with the lowest particle size of 0.425 mm tends to produce aqueous extracts of tea, ginger, and tea–ginger with highest antioxidant content. At this lowest particle size all the antioxidant properties assayed were maximized. The TPC of aqueous extracts obtained from the 0.425 mm tea, ginger, and tea–ginger powders were 685.44 ± 175, 283.58 ± 19, and 483.02 ± 176 mg gallic acid equivalent (GAE) L^−1^, respectively. The TPC of aqueous extracts obtained from the 0.710 mm tea, ginger, and tea–ginger powders were 679.06 ± 169, 208.94 ± 147, and 400.10 ± 130 mg GAE L^−1^, respectively. However, for the aqueous ethanolic and ethanolic extracts, the particle size that maximized the antioxidant extraction varied depending on the antioxidant property that was being assayed. The study suggests that particle size influences the extraction of antioxidants. Also, the optimum powder size that would maximize antioxidant extraction is dependent on the solvent used and the antioxidant property being measured.

## 1 INTRODUCTION

Tea (*Camellia sinensis*) and ginger (*Zingiber officinale*) are plants with health benefits. The research on the influence of tea on human health has been driven by the growing need to provide naturally healthy diets that include plant‐derived polyphenols (Karori, Wachira, Wanyoko, & Ngure, 2007). The consumption of tea flavonoid has been linked to lower incidences of chronic diseases such as cardiovascular disease and cancer (Rusak, Komes, Likic′, Horzˇic′, & Kovac, 2008). Aqueous solvents of black tea extracts have also been shown to possess antibacterial activity against some foodborne pathogens (Turkmen, Velioglu, Sari, & Polat, 2007). Ginger is also known to possess anti‐inflammatory, antinausea, anticarcinogenic, and antioxidant effects (Bode & Dong, 2011). The combination of tea and ginger has also been reported to possess synergistic antioxidant effect (Makanjuola, Enujiugha, Omoba, & Sanni, 2015a).

Parameters that have high influence on the amount and composition of antioxidants in extracts include the extraction solvent, temperature, extraction time (duration), solvent‐to‐solid ratio, and storage conditions (Michiels, Kevers, Pincemail, Defraigne, & Jacques Dommes, 2012). Extraction of antioxidants from tea, ginger, and their blends have been reported to be affected by extraction temperature, powder concentration, extraction time, pH, solvent type, and solvent concentration (Makanjuola, Enujiugha, & Omoba, 2016; Makanjuola, Enujiugha, Omoba, & Sanni, 2015b,c; Makanjuola et al., 2015a; Perva‐Uzunalic et al., 2006; Savić et al., 2013; Turkmen et al., 2007; Vuong, Golding, Stathopoulos, Nguyen, & Roach, 2011).

Available studies on the effect of particle size on tea antioxidant extraction were performed using aqueous extraction. However it would also be useful to study the effect of particle size on the ethanolic extraction of tea—as this can present some advantages with respect to preparation of tea nutraceuticals—as the ethanolic solvent could enhance the extraction of some polyphenols that cannot be extracted by aqueous extraction. Castiglioni, Damiani, Astolfi, and Carloni (2015) also studied the total phenol content, total flavonoid content, and antioxidant activity of whole and ground tea leaves when extracted with water—and they reported that these properties were higher for the milled tea. Some studies available on ginger particle size had to do with its effect on extraction of ginger oil (Chen, Chung, Wang, & Huang, 2011)—and the influence of superfine grinding on physical–chemical properties of the powder (Zhao, Yang, Gai, & Yang, 2009). This present study looked at the influence of particle size and extraction solvent on antioxidant contents of black tea, ginger, and tea–ginger extracts. The solvents used for extraction were water, ethanol, and aqueous ethanol.

## 2 MATERIALS AND METHODS

### 2.1 Powder preparation

Tea leaves were harvested from Obudu Mountain in Cross River State and the ginger rhizomes were obtained from Kaduna State, both in Nigeria. Tea leaves were processed into black tea by allowing them to wilt, oxidize, and dry in the sun, and the dried tea leaves were then ground. The ginger rhizomes were peeled, sun‐dried, and ground. The powders were passed through sieves of the following sizes: 0. 425, 0.750, and 1.18 mm, and the powder retained on the sieves were used for further analysis. Three powder sizes were used for the tea extraction (Table 1). Two powder sizes were used for the ginger and tea–ginger (blend) extraction (Table 1) because powder with particle size greater than 1.18 mm was not obtained for ginger during the sieve analysis.

**Table 1:** Powder samples and solvents used to evaluate particle size effect on antioxidant extraction

Powder sample	Powder size (mm)	Extraction solvents
Tea	0.425, 0.710,1.180	Water, absolute ethanol, aqueous ethanol (v/v)
Ginger	0.425, 0.710	Water, absolute ethanol, aqueous ethanol (v/v)
Tea–ginger blend (2:1)	0.425, 0.710	Water, absolute ethanol

0.425 mm powder size (0.425 ≥ x < 0.710), 0.710 mm powder size (0.710 ≥ x < 1.180), 1.180 mm powder size (≥1.180).

### 2.2 Powder extraction

The tea, ginger, and tea–ginger (2:1) powders were extracted under multiresponse optimized conditions determined in the laboratory. These optimization conditions were determined using the desirability concept. The multiresponse optimization was set to maximize the following antioxidant properties: DPPH radical scavenging activity (DPPH), ABTS radical scavenging activity (ABTS), TPC, iron chelating activity (ICA), total flavonoid content (TFC), and peroxide scavenging activity (PSA). Some of these optimization results have been reported in Makanjuola et al. (2015b,c, 2016). These extraction (multiresponse optimization) conditions used for the different powders are shown in Table 2.

**Table 2:** Extraction conditions

Treatment	Temperature (°C)	Concentration (g 100 ml^−1^)	Time (min)
Aqueous ginger extraction	96.00	2.10	90.00
Aqueous tea extraction	54.00	2.10	88.00
Aqueous ethanolic (50% v/v) ginger extraction	30.00	1.90	91.00
Aqueous ethanolic (50% v/v) tea extraction	30.00	1.75	5.00
Absolute ethanolic ginger extraction	44.00	2.10	47.00
Absolute ethanolic tea extraction	66.00	0.80	90.00
Aqueous tea–ginger 2:1 extraction	96.00	1.68	90.00
Ethanolic tea–ginger 2:1 extraction	50.00	2.10	5.00

The tea and ginger powders were extracted as described in Makanjuola et al. (2015b). Extraction was done in a conical flask placed on temperature‐controlled magnetic stirrer (UC 152; Bibby Scientific, Staffordshire, UK). The flask was covered with aluminum foil to prevent light penetration. To guarantee the accuracy of the extraction temperature, a temperature controller (SCT 1, Bibby Scientific, Staffordshire, UK) was placed inside the conical flask and connected to the temperature‐controlled magnetic stirrer. When the required extraction temperature was reached, the required weight of powder was introduced into the conical flask.

### 2.3 Antioxidant analysis

Extracts were analyzed for DPPH radical scavenging activity, ABTS radical scavenging activity, total phenol content, iron chelating activity, total flavonoid content, and peroxide scavenging activity.

### 2.4 ABTS radical scavenging activity

ABTS was assayed using the method of Miliauskas, Venskutonis, and van Beek (2004), as described in Spradling (2008). ABTS stock solution was prepared by mixing 44.8 mg of ABTS, 8.12 mg potassium persulfate, and 20 ml of distilled water. The solution was stored in the dark for 12 hr. The ABTS working solution was also prepared by mixing 5 ml of the ABTS stock solution with 145 ml of phosphate buffer solution. One hundred microliter of each extract or standard was added to 2,900 μl of the ABTS working solution and kept for 15 min before reading spectrophotometrically at 734 nm against a blank solution. Trolox was used as standard and result was expressed as milligram Trolox equivalent per liter (mg TE L^−1^).

### 2.5 DPPH radical scavenging activity

DPPH was assayed using the method of Sompong, Siebenhandl‐Ehn, Linsberger‐Martina, and Berghofer (2011). The reaction mixture consisted of 1.5 ml DPPH working solution and 300 μl extract. The mixture was shaken and left to stand for 40 min in the dark at room temperature. Absorbance was read at 515 nm relative to a control.

### 2.6 Iron chelating activity

Iron chelating activity was evaluated as described in Ozena, Demirtas, and Aksit (2011). The samples were added to a solution of 2 mmol L^−1^ FeCl_2_ (0.05 ml). The reaction was initiated by the addition of 5 mmol L^−1^ ferrozine (0.2 ml) and the mixture was incubated at room temperature for 10 min. The absorbance was read at 562 nm.

### 2.7 Peroxide scavenging activity

Peroxide scavenging activity was assayed using the method of Smirnoff and Cumbes (1989) as explained in Ozena et al. (2011). Peroxide radicals were produced by mixing of H_2_O_2_ and FeSO_4_. The reaction mixture consisted of 1 ml FeSO_4_ (1.5 mmol L^−1^), 0.7 ml H_2_O_2_ (6 mmol L^−1^), 0.3 ml sodium salicylate (20 mmol L^−1^), and appropriate volume of extracts. This mixture was stored for 1 hr at room temperature. Absorbance of the hydroxylated salicylate complex was measured at 562 nm.

### 2.8 Total flavonoid content

Total flavonoid content was determined as described by Prommuaka, De‐Eknamkulb, and Shotipruka (2008). A 0.5 ml of catechin solutions or the extracted samples was added to 1.5 ml of 95% ethanol (v/v), 0.1 ml of 10% aluminum chloride (AlCl_3_·6H_2_O) (m/v), 0.1 ml of 1 mol L^−1^ of potassium acetate, and 2.8 ml of distilled water. The mixture was incubated at room temperature for 30 min and the absorbance of the mixture was measured against a blank using a spectrophotometer at 415 nm. Catechin was used as standard and result was expressed as milligram catechin equivalent per liter (mg CE L^−1^).

### 2.9 Total phenol content

Total phenol content was determined as described by Waterhouse (2003), using the method of Slinkard and Singleton (1977). A 50 μl sample of the calibration solution, blank, or extract, was added to 1.58 ml water, and 100 μl of Folin–Ciocalteu reagent. After 8 min, 300 μl of sodium carbonate solution was added. The solutions were left at room temperature for 1 hr and absorbance of each solution was determined at 765 nm against a blank. Gallic acid was used as standard and result was expressed as milligram catechin equivalent per liter (mg GAE L^−1^).

### 2.10 Statistical analysis

The data were analyzed using analysis of variance and Fisher's least significant difference using XLSTAT Pro, 2013 (Addinsoft, Paris, France).

## 3 RESULTS

### 3.1 Antioxidant properties of aqueous extracts

Tea particle size of 0.425 mm produced extract with highest total phenol content and DPPH radical scavenging activity (Table 3). The particle sizes 0.425 mm and 0.710 mm produced extracts with the highest total flavonoid content (12,711.11 and 12,002.78 mg CE L^−1^, respectively), highest ABTS radical scavenging activity (0.903 and 0.890 mg TE L^−1^, respectively), and highest peroxide scavenging activity (61.80% and 60.82%, respectively). No significant differences (*p* < .05) were observed between the particle sizes (0.425 mm and 0.710 mm) for total flavonoid content, ABTS radical scavenging activity, peroxide scavenging activity, and DPPH radical scavenging activity (Table 3) for ginger powder extraction. The total flavonoid content for the 0.425 mm and 0.710 mm ginger powder extracts were 2,083.05 and 2,655.56 mg CE L^−1^, respectively. The DPPH radical scavenging activity for the 0.425 and 0.710 mm ginger powder extracts were 95.98% and 96.64%, respectively. The powder size 0.425 mm had the highest iron chelating activity (84.48%) when compared with powder size of 0.710 mm, which had an iron chelating activity of 74.53% (Table 3). The ginger powder with 0.710 mm particle size had a lower total phenol content of 208.94 GAE L^−1^ when compared the ginger powder with particle size of 0.425 mm, which had a total phenol content of 283.58 GAE L^−1^. No significant differences (*p* < .05) were observed between the particle sizes (0.425 mm and 0.710 mm) for total flavonoid content, peroxide scavenging activity, iron chelating activity, and DPPH radical scavenging activity of tea–ginger extract (Table 3). The total flavonoid content, peroxide scavenging activity, iron chelating activity, and DPPH radical scavenging activity of the 0.425 mm tea–ginger powder were 8,975.00 mg CE L^−1^, 74.84%, 87.13%, and 97.84%, respectively. The total flavonoid content, peroxide scavenging activity, iron chelating activity, and DPPH radical scavenging activity of the 0.710 mm tea–ginger powder were 7,954.17 mg CE L^−1^, 71.09%, 85.61%, and 97.59%, respectively (Table 3). Total phenol content and ABTS radical scavenging activity was higher for the 0.425 mm tea–ginger powder than the 0.710 mm tea–ginger powder. The total phenol content and ABTS radical scavenging activity of the 0.425 mm tea–ginger powder were 483.02 mg GAE L^−1^ and 0.922 mg TE L^−1^, respectively. The total phenol content and ABTS radical scavenging activity of the 0.710 mm tea–ginger powder were 400.10 mg GAE L^−1^ and 0.900 TE L^−1^, respectively.

**Table 3:** Influence of particle size on antioxidant properties of aqueous extracts

Particle size (mm)	Total flavonoid content (mg CE L^−1^)	Total phenol content (mg GAE L^−1^)	ABTS (mg TE L^−1^)	Peroxide scavenging activity (%)	Iron chelating activity (%)	DPPH (%)
*Tea*						
0.425	12,711.11 ± 3,980 a^a^	685.44 ± 175 a	0.903 ± 0.032 a	61.80 ± 10 a	85.82 ± 6 a	97.53 ± 0.70 a
0.710	12,002.78 ± 4,335 ab	679.06 ± 169 b	0.890 ± 0.042 a	60.82 ± 4 a	84.03 ± 8 b	96.25 ± 2 b
1.180	9,641.67 ± 2,456 b	458.68 ± 88 c	0.755 ± 0.22 b	55.18 ± 11 b	86.29 ± 3 a	96.67 ± 2 b
*Aqueous ginger*						
0.425	2,083.05 ± 1,428 a^a^	283.58 ± 19 a	0.932 ± 0.035 a	69.32 ± 4 a	84.48 ± 16 a	95.98 ± 2 a
0.710	2,655.56 ± 1,627 a	208.94 ± 147 b	0.940 ± 0.041 a	68.36 ± 10 a	74.53 ± 9 b	96.64 ± 2 a
*Aqueous tea–ginger*						
0.425	8,975.00 ± 2,361 a^a^	483.02 ± 176 a	0.922 ± 0.033 a	74.84 ± 0.033 a	87.13 ± 10 a	97.84 ± 1 a
0.710	7,954.17 ± 539 a	400. 10 ± 130 b	0.900 ± 0.025 b	71.09 ± 8 a	85.61 ± 4 a	97.59 ± 0.88 a

a Mean ± *SD* values in the same column and row heading followed by the same alphabet are not significantly different (*p* > .05) LSD. Data presented as mean of triplicate measurement.

### 3.2 Antioxidant properties of absolute ethanolic extracts

The ethanolic extract of 0.425 mm tea powder had lower total flavonoid content and lower total phenol content when compared to the ethanolic extract of 0.710 mm tea powder (Table 4). The ethanolic extract of 1.180 mm tea powder had significantly lower (*p* < .05) total phenol content and ABTS radical scavenging activity when compared to the ethanolic extracts of the 0.425 mm and 0.710 mm tea powders. The 1.180 mm tea powder ethanolic extract had the highest iron chelating activity of 83.00%, while the 0.710 mm tea powder ethanolic extract had the lowest iron chelating activity of 72.99%. No significant difference was observed in the DPPH radical scavenging activity of all the tea powder extracts. The DPPH radical scavenging activity of the ethanolic tea powder extracts were 97.27%, 96.75%, and 97.42% for the 0.425 mm, 0.710 mm, and 1.180 mm powders, respectively. For the ethanolic ginger extraction, the 0.710 mm ginger powder extract had higher total phenol content and iron chelating activity (281 GAE L^−1^ and 84.63%, respectively) than the 0.425 mm ginger powder (260.88 GAE L^−1^ and 75.39%, respectively). No significant differences (*p* > .05) were found between the two ginger powder extracts in their total flavonoid content, ABTS radical scavenging activity, peroxide scavenging activity, and DPPH radical scavenging activity (Table 4). Also in the ethanolic tea–ginger extraction, no significant differences were observed between the two powder sizes in their total flavonoid content, ABTS radical scavenging activity, peroxide scavenging activity, and DPPH radical scavenging activity. However, the 0.425 mm tea–ginger powder had higher total phenol content and iron chelating activity (322.31 GAE L^−1^ and 91.53%, respectively) than the 0.710 mm tea–ginger powder (281.24 GAE L^−1^ and 89.48%, respectively).

**Table 4:** Influence of particle size on antioxidant properties of absolute ethanolic extracts

Particle size (mm)	Total flavonoid content (mg CE L^−1^)	Total phenol content (mg GAE L^−1^)	ABTS (mg TE L^−1^)	Peroxide scavenging activity (%)	Iron chelating activity (%)	DPPH (%)
*Tea*						
0.425	10,183.33 ± 1,593 b	453.26 ± 0.83b	0.938 ± 0.02 a	50.55 ± 4.46 ab	77.72 ± 1.16 b	97.27 ± 0.81 a
0.710	16,641.66 ± 3,843 a	466.12 ± 1.83 a	0.838 ± 0.02 b	56.90 ± 2.96 a	72.99 ± 0.32 c	96.75 ± 0.83 a
1.180	12,225.00 ± 2,707 ab	370.17 ± 4.32 c	0.464 ± 0.05 c	42.37 ± 5.05 b	83.00 ± 1.68 a	97.42 ± 0.19 a
*Ginger*						
0.425	3,850.00 ± 433 a	260.88 ± 9.19 b	0.908 ± 0.02 a	72.77 ± 2.94 a	75.39 ± 1.00 b	95.64 ± 0.87 a
0.710	3,350.00 ± 573 a	281.71 ± 5.85 a	0.923 ± 0.02 a	79.49 ± 9.00 a	84.63 ± 1.42 a	97.47 ± 1.41 a
*Tea–ginger*						
0.425	8,850.00 ± 573 a	322.31 ± 3.47 a	0.951 ± 0.02 a	83.76 ± 3.81 a	91.53 ± 0.67 a	98.72 ± 0.50 a
0.710	8,183.33 ± 711 a	281.24 ± 3.52 b	0.883 ± 0.02 b	77.66 ± 3.53 a	89.48 ± 0.63 b	98.35 ± 0.31 a

Mean ± *SD* values in the same column and row heading followed by the same alphabet are not significantly different (*p* > .05) LSD. Data presented as mean of triplicate measurement.

### 3.3 Antioxidant properties of aqueous ethanolic extracts

The aqueous ethanolic extract of the 0.710 mm tea powder had the highest total phenol content when compared with the 0.425 mm and 1.180 mm tea powder (Table 5). The 0.425 mm tea powder had the highest iron chelating activity (90.78%) when compared to the 0.710 mm and 1.18 mm tea powders (88.86% and 86.91%, respectively). The 0.425 mm and 0.710 mm tea powders both had higher peroxide scavenging activity than the 1.180 mm tea powder. No significant differences (*p* > .05) were observed in the total flavonoid contents and the DPPH radical scavenging activities of the aqueous ethanolic extracts for the three tea powder sizes (Table 5). The aqueous ethanolic extract of the 0.710 mm ginger powder had higher total phenol content than the aqueous ethanolic extract of the 0.425 mm ginger powder (Table 5). No significant differences were observed in all the other antioxidant properties that were determined for the 0.425 mm and 0.710 mm aqueous ethanolic ginger powder extracts.

**Table 5:** Influence of particle size on antioxidant properties of aqueous ethanolic extracts

Particle size (mm)	Total flavonoid content (mg CE L^−1^)	Total phenol content (mg GAE L^−1^)	ABTS (mg TE L^−1^)	Peroxide scavenging activity (%)	Iron chelating activity (%)	DPPH (%)
*Tea*						
0.425	12,308.33 ± 5,160 a	823.38 ± 6.49 b	0.874 ± 0.02 b	62.79 ± 2.78 a	90.78 ± 0.40 a	98.19 ± 1.05 a
0.710	8,475.00 ± 2,103 a	849.81 ± 3.12 a	0.907 ± 0.01 a	63.03 ± 2.75 a	88.86 ± 0.66 b	97.84 ± 0.44 a
1.180	8,016.67 ± 439 a	564.69 ± 7.76 c	0.876 ± 0.01 ab	55.64 ± 0.63 b	86.91 ± 0.84 c	98.19 ± 1.51 a
*Ginger*						
0.425	1,058.33 ± 315 a	301.95 ± 3.38 b	0.926 ± 0.05 a	64.49 ± 2.07 a	92.96 ± 1.15 a	98.13 ± 0.50 a
0.710	891.67 ± 315 a	330.64 ± 6.56 a	0.926 ± 0.06 a	58.55 ± 0.63 b	94.67 ± 0.91 a	98.42 ± 0.52 a

Mean ± *SD* values in the same column and row heading followed by the same alphabet are not significantly different (*p* > .05) LSD. Data presented as mean of triplicate measurement.

## 4 DISCUSSION

The present study indicated that particle size has an influence on the antioxidant content of tea, ginger and tea–ginger extracts. For the aqueous extraction, the tea, ginger and tea–ginger powders with the lower particle sizes tend to produce extracts with higher antioxidant properties. At the lowest particle size of 0.425 mm, all the antioxidant properties investigated were maximized for the aqueous extraction of tea, ginger, and tea–ginger powders (Table 6). Size reduction of plant substrates before extraction maximizes the surface area, which in turn enhances the mass transfer of active principle from plant material to the solvent (Handa, 2008). Wang and Helliwell (2001) have reported that content of myricetin, quercetin, and kaempferol in ground tea samples was 36% higher than that in unground ones (whole leaf). Green tea with particle sizes of 0.25–1 mm have also been reported to yield higher catechins than tea with particle sizes >1 mm (Vuong et al., 2011).

**Table 6:** Percentage of assayed antioxidant properties maximized by the different particle sizes

Powder	Aqueous extraction	Ethanolic extraction	Aqueous ethanolic extraction
*Tea*			
0.425 mm	100.00%^a^	50.00%	66.67%
0.710 mm	50.00%	66.67%	83.33%
1.180 mm	16.67%	50.00%	33.33%
*Ginger*			
0.425 mm	100.00%	66.67%	83.33%
0.710 mm	66.67%	100.00%	83.33%
*Tea–ginger*			
0.425 mm	100.00%	100.00%	—
0.710 mm	66.67%	50.00%	—

a Percentage score was calculated from the number of times the extract from a particle size produced highest antioxidant values—for a particular solvent extraction. Hence a score of 100% means that aqueous extracts obtained from the 0.425 mm tea powder scored highest on all the six antioxidant assays when compared to the other aqueous tea extracts from the 0.710 mm and 1.180 mm powder.

Unlike the aqueous extraction, not a single particle size was able to maximize all the antioxidant properties for the aqueous ethanolic and ethanolic extractions (Table 6). It was observed that the particle size that maximized the antioxidant extractions varied between the different powder sizes—depending on the antioxidant property that was being measured. For example, during the aqueous ethanolic extraction of tea powder, the 0.425 mm powder was only able to maximize four of the six (66.67%) antioxidant properties assayed (Table 6). The 0.710 mm powder and 1.180 mm powder were only able to maximize five (83.33%) and two (33.33%) of the six antioxidant properties assayed, respectively.

These observations brought to fore the following points:

(1) The optimum particle size (size that maximizes antioxidant property) is solvent dependent.
(2) The optimum particle size is also dependent on the antioxidant properties being measured.
(3) The lowest particle size may not always give the highest antioxidant property.

Although it is a known fact that a reduction in particle size could always lead to increased extraction efficiency, however, a critical particle size is reached such that any further reduction in the particle size—could lead to no further increase—or a reduction—in extraction efficiency. This point is also justified by the study of Brewer, Kubola, Siriamornpun, Herald, and Shi (2014), where the influence of particle size on antioxidant properties (diphenylpicrylhydrazyl radical scavenging activity, ferric reducing/antioxidant power [FRAP] assay, oxygen radical absorbance capacity [ORAC]) and total antioxidant capacity) of unmilled whole bran (coarse treatment) were compared to whole bran milled to medium and fine treatments from the same wheat bran. It was observed that the coarse treatment exhibited significantly higher antioxidant properties compared to the fine treatment; except for the ORAC value, in which the coarse extract was significantly lower. Zhang et al. (2016) reported that no significant difference was found in the total phenol content of water extract of superfine black tea powder (13.67 μm) and coarse tea powders (228.67, 161.00, 140.67, 79.07 μm). According to Vuong et al. (2011), the extraction of catechins may be impaired when brewing very small particle sizes because these small particles may settle to the bottom and, like sand, form sediments at the bottom of the extraction container, which could reduce the flow‐through of water and, therefore, the tea would not effectively interact with the water. It should also be noted that a very small powder particle size may become slimy during extraction and create difficulty during filtration.

## 5 CONCLUSION

Particle size has been shown to play a role in the extraction of antioxidants from tea, ginger, and their blend. While all the antioxidant properties of the aqueous extract tend to increase as the particle size is reduced from 1.180 to 0.425 mm, it was observed that for the ethanolic and aqueous ethanolic extracts, the particle size that maximizes the antioxidant extraction varied—depending on the antioxidant property been assayed. The study suggests that though a reduction in particle size could enhance the extraction of antioxidants from tea and ginger; a critical particle size is reached whereby a further reduction in particle size could impair the extraction of antioxidants.

## CONFLICT OF INTEREST

Conflict of interest does not exist in this study.
